# Genomic insights into virulence, antimicrobial resistance, and adaptation acumen of *Escherichia coli* isolated from an urban environment

**DOI:** 10.1128/mbio.03545-23

**Published:** 2024-02-20

**Authors:** Poorvi Saini, Viraj Bandsode, Anuradha Singh, Suresh Kumar Mendem, Torsten Semmler, Munirul Alam, Niyaz Ahmed

**Affiliations:** 1 Department of Biotechnology and Bioinformatics, Pathogen Biology Laboratory, University of Hyderabad, Hyderabad, Telangana State, India; 2 Robert Koch Institute, Berlin, Germany; 3 International Centre for Diarrheal Disease Research, Bangladesh (icddr,b), Dhaka, Bangladesh; Cornell University, Ithaca, New York, USA

**Keywords:** *Escherichia coli*, genome analysis, virulence, antimicrobial resistance, environment

## Abstract

Populations of common commensal bacteria such as *Escherichia coli* undergo genetic changes by the acquisition of certain virulence and antimicrobial resistance (AMR) encoding genetic elements leading to the emergence of pathogenic strains capable of surviving in the previously uninhabited or protected niches. These bacteria are also reported to be prevalent in the environment where they survive by adopting various recombination strategies to counter microflora of the soil and water, under constant selection pressure(s). In this study, we performed molecular characterization, phenotypic AMR analysis, and whole genome sequencing (WGS) of *E. coli* (*n* = 37) isolated from soil and surface water representing the urban and peri-urban areas. The primary aim of this study was to understand the genetic architecture and pathogenic acumen exhibited by environmental *E. coli*. WGS-based analysis entailing resistome and virulome profiling indicated the presence of various virulence (adherence, iron uptake, and toxins) and AMR encoding genes, including *bla*_NDM-5_ in the environmental isolates. A majority of our isolates belonged to phylogroup B1 (73%). A few isolates in our collection were of sequence type(s) (ST) 58 and 224 that could have emerged recently as clonal lineages and might pose risk of infection/transmission. Mobile genetic elements (MGEs) such as plasmids (predominantly) of the IncF family, prophages, pipolins, and insertion elements such as IS1 and IS5 were also observed to exist, which may presumably aid in the propagation of genes encoding resistance against antimicrobial drugs. The observed high prevalence of MGEs associated with multidrug resistance in pathogenic *E. coli* isolates belonging to the phylogroup B1 underscores the need for extended surveillance to keep track of and prevent the transmission of the bacterium to certain vulnerable human and animal populations.

**IMPORTANCE:**

Evolutionary patterns of *E. coli* bacteria convey that they evolve into highly pathogenic forms by acquiring fitness advantages, such as AMR, and various virulence factors through the horizontal gene transfer (HGT)-mediated acquisition of MGEs. However, limited research on the genetic profiles of environmental *E. coli*, particularly from India, hinders our understanding of their transition to pathogenic forms and impedes the adoption of a comprehensive approach to address the connection between environmentally dwelling *E. coli* populations and human and veterinary public health. This study focuses on high-resolution genomic analysis of the environmental *E. coli* isolates aiming to understand the genetic similarities and differences among isolates from different environmental niches and uncover the survival strategies employed by these bacteria to thrive in their surroundings. Our approach involved molecular characterization of environmental samples using PCR-based DNA fingerprinting and subsequent WGS analysis. This multidisciplinary approach is likely to provide valuable insights into the understanding of any potential spill-over to human and animal populations and locales. Investigating these environmental isolates has significant potential for developing epidemiological strategies against transmission and understanding niche-specific evolutionary patterns.

## INTRODUCTION

One Health inspired research, through numerous studies, has identified *Escherichia coli* as a priority pathogen with varied virulence or contaminating potential towards humans, animals, food, and environmental niches such as water and soil. In addition to colonizing the human gut within a few hours of birth, it is also capable of potentially interacting with its host that ranges from benign commensalism to severe forms of pathogenicity - thus becoming one of the most frequent causal agents of bacterial infections globally (1). Despite being a commensal gut bacterium, *E. coli* is thought to be a reservoir of acquired antimicrobial resistance (AMR) determinants (2). Previously, it was believed that *E. coli* cannot survive well outside the host because of its inability to multiply under environmental conditions (3). However, subsequent studies revealed that *E. coli* can survive outside its host for extended periods of time with a significant prevalence in soil, sand, and silt in tropical, subtropical, and temperate regions along with some water bodies (4). Due to their capacity to adapt to environmental flux through phenotypic and genetic plasticity, *E. coli* are the most prevalent intestinal bacteria in the environment. Such versatility enabling their survival in the environmental niches can be attributed to the presence of vital nutrients including organic carbon, phosphate, and nitrogen as well as the capacity to cope with nutritional challenges or deficiencies (5). Over the course of evolution, *E. coli* has acquired many genotypic and phenotypic traits, enabling them to quickly adapt to environmental fluctuations. These traits include (i) activation of enzymes to catabolize available nutrients, (ii) toxin synthesis restricting the invasion by other organisms within the environment, (iii) switching to a survival state facilitating stress tolerance, keeping them viable even with nutrient deprivation, and (iv) expression of various antibiotic resistance genes and virulence factors, collectively contributing towards its evolution into a seasoned pathogenic agent (6, 7). Under environmental conditions, the growth of the bacterium is influenced by both biotic and abiotic factors. Abiotic factors such as temperature, availability of nutrients, pH, and solar radiation impact the proliferation of bacteria in environmental niches (8). The capacity of *E. coli* to utilize resources, interact with other microbes to outgrow, and build biofilms in natural settings is an example of biotic factors (9). Because of the aforementioned environmental factors and stress conditions, pathogenic bacteria may undergo evolutionary changes leading to the transition from low virulence to high virulence during this period (10). Genetic drivers of such resilience could be acquired or transmitted through HGT, which can propagate these traits and adorn the recipient organisms with various fitness advantages (11). The genome evolution occurs due to genetic alterations that can take place by three common mechanisms: (i) gene acquisition by HGT; (ii) gene deletions, rearrangements, and point mutations; and (iii) gene duplication followed by amplification and genome decay, which can occur through HGT (11). MGEs such as plasmids, prophages, and insertion elements also play an important role in transferring genetic material through HGT.

The emergence of AMR in *E. coli* has become a prominent issue of concern, manifesting with increased frequency in both human and veterinary medicine sectors, worldwide (12). Genomic analyses of human commensals and environmental bacteria indicate the presence of a substantial amount of resistance determinants within their genomes, which were not acquired through horizontal transmission and existed prior to the clinical application of antibiotics. Still, AMR is largely considered as an outcome of various anthropogenic and therapeutic activities operating in a complex fashion. This happens both within clinical and environmental arenas with the major involvement of diverse resistance genes that are known to exist within the gut microbiota (13). On a global scale, the prevalence and dissemination of antibiotic-resistant *E. coli* are recognized as a significant concern in the context of both human and animal populations. To address this complex issue, a comprehensive One Health approach is essential. This approach recognizes the interconnectedness of human, animal, and environmental health and emphasizes collaboration between disciplines to combat the growing threat of AMR and its associated pathogenic risks in urban environments. Another known element influencing the persistence of *E. coli* in natural habitats is biofilms generated by the organism on surfaces in different environments, such as sediments (14). The bacteria are shielded by biofilms from harmful environmental factors such as UV radiation, desiccation, protozoan predators, and chemicals like antibiotics and detergents (15). The development of biofilm contributes to prolonged microbial survival in environmental settings. Biofilms facilitate and amplify resistance to multiple antibiotics due to various factors, such as limited diffusion of antimicrobial agents through the biofilm matrix, reduced interaction of antimicrobial agents with the biofilm-forming bacteria, enzyme-mediated resistance, changes in cellular metabolism levels within the biofilm, and adaptations of the outer membrane structures (16). Recent research has unveiled the prevalence of multidrug-resistant (MDR) *E. coli* in poultry and farm animals, as indicated by some studies (17, 18), and highlighted the associated risks of transmission from such sources (19). Moreover, freshwater bodies across the globe have been found to harbor MDR *E. coli* bacteria (20–22). Some studies have even detected such strains in drinking water (23). The existence of these pathogenic isolates within the environmental settings of urban regions presents a significant challenge to human health. There have been limited investigations into the genetic patterns of environmental *E. coli*, specifically in India, thus hindering our understanding of their potential transformation into pathogenic forms. Exploring these environmental isolates holds promise for devising preventive measures against transmission.

Molecular characterization of environmental isolates may unravel the acquired characteristics and serve as a basis for comparison between clinical pathogenic and environmental isolates. Understanding the molecular diversity, clonal lineages, and phylogeny of the bacteria has been significantly assisted by a variety of subtyping methods for *E. coli* with various approaches and specificities (24–26). In addition to traditional serotyping, which uses antibodies to identify the surface antigens O, H, and K (27), WGS as well as PCR-based methods allow for quicker and more accurate classification of the serotypes (28). PCR-based methods are simpler to produce results rapidly for initial confirmation. Here, we employed different PCR-based fingerprinting methods [based on enterobacterial repetitive intergenic consensus (ERIC), repetitive extragenic palindromic (REP) sequences, and randomly amplified polymorphic DNA (RAPD)] for the preliminary analysis of the isolates that were further confirmed and validated by WGS to understand the diversity and characteristics of isolates obtained from the environment. WGS can provide an enhanced resolution to the pathogenic attributes exhibited by bacteria. *In silico* resistome and virulome profiling studies were performed to understand the prevalence patterns of virulence and AMR encoding genes. The occurrence of HGT events has been summarized by predicting mobile genetic elements such as plasmids, prophages, and insertion elements that may mobilize or shuffle/shuttle the resistance or virulence encoding genes. The phylogenetic relatedness of the isolates has been documented based on pan genome analysis and orthologous groups.

We believe our multifaceted analyses provide essential insights into the diversity and survival of *E. coli* bacteria in the environment and their transmission as well as virulence attributes in the context of AMR and One Health.

## RESULTS

### Bacterial isolates, phylogroups, and antibiotic sensitivity (AST) profiles

**Fig 1:**
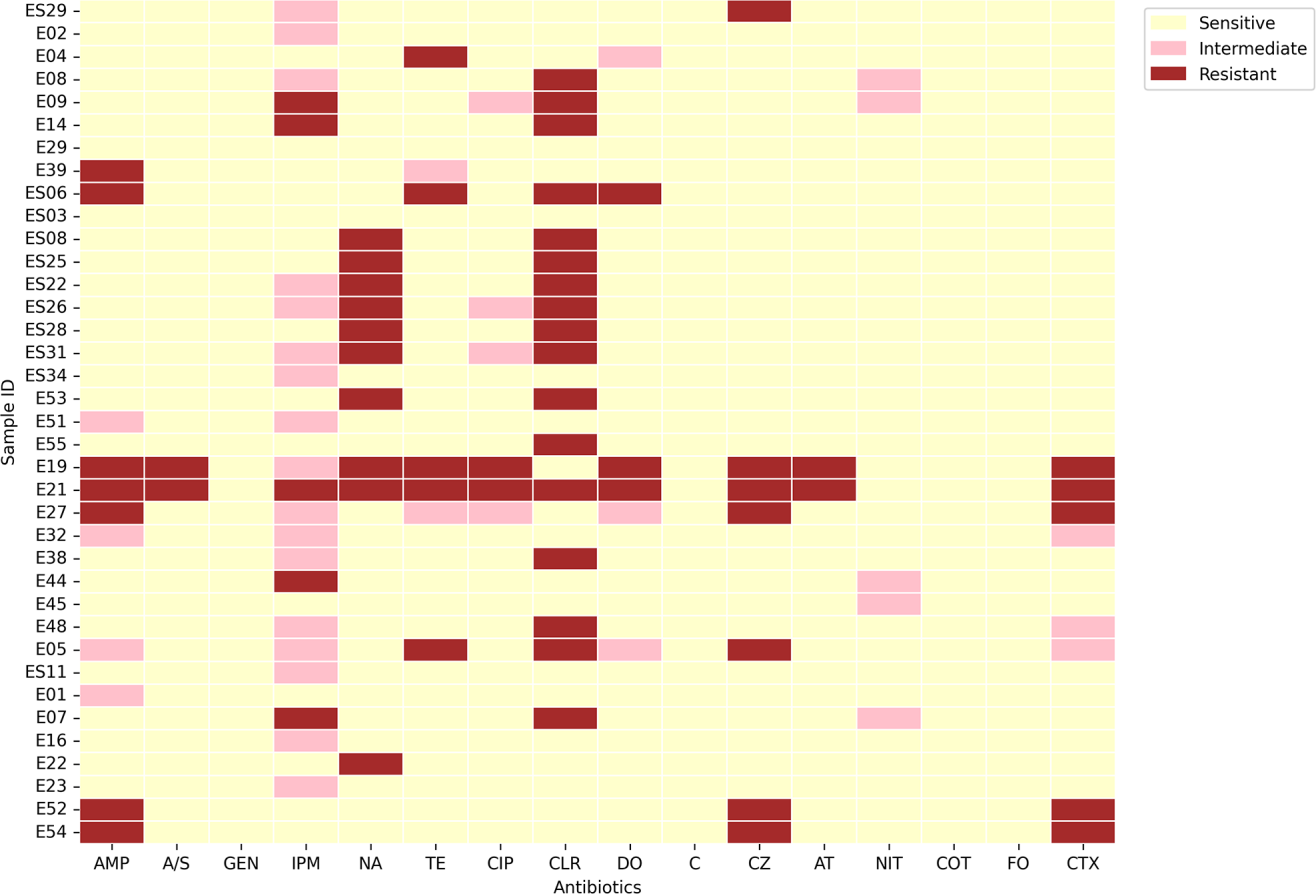
Heatmap depicting resistance profiles of the isolates obtained by experimental analysis using the Kirby–Bauer disc diffusion assay. The isolates are defined as resistant, intermediate, and susceptible for each antimicrobial drug according to the Clinical & Laboratory Standards Institute (CLSI, 2021) guidelines. Yellow color indicates sensitive, and pink color indicates intermediate while red color indicates resistance to respective antibiotics.

A total of 37 *E. coli* isolates were obtained out of 100 environmental samples that were collected, screened, and subsequently plated on MacConkey and EMB agar plates. Out of these, 7 isolates were from community service areas, 14 from lakes, 5 from sewage water, and 11 from urban slum areas (Table 1). IMViC procedures displayed positive for indole and methyl red tests while negative for Voges Proskauer and citrate utilization tests. Predominantly, isolates in this study belonged to phylogroup B1 (27 out of 37), while others were associated with phylogroup A, B2, and E (less than 20% of the isolates). All the isolates were screened against 16 different antibiotics according to the disk diffusion protocol. The AST revealed that most of them were resistant to at least 1 of the 16 antibiotics. Maximum resistance was observed against clarithromycin (45.9%) followed by nalidixic acid (27.02%). These environmental isolates were observed to demonstrate less resistance to ampicillin/sulbactam (5.40%), ciprofloxacin (5.40%), aztreonam (5.40%), and doxycycline (8.10%). The isolates were completely sensitive to co-trimoxazole, fosfomycin, chloramphenicol, and gentamicin while many of the isolates showed intermediate resistance to imipenem (45.94%) (Fig. 1).

**TABLE 1:** Geographical coordinates of the isolates along with assembly statistics of their genomes (GC%, total genome length, number of contigs, and N50 value)

Isolates	Collection date (yr-mo-day)	Geographical location	Isolation source	Latitude and longitude	Genome accession	GC%	Total length	No. of contigs	N50
E02	2022-09-02	India: Hyderabad	Environment: water	17.374402°N, 78.470938°E	JAVALI000000000	51.08	4,467,148	237	40,233
E05	2022-09-02	India: Hyderabad	Environment: water	17.374815°N, 78.469746°E	JAVALH000000000	51.02	4,858,372	287	33,328
E16	2022-09-02	India: Hyderabad	Environment: water	17.41300°N, 78.472672°E	JAVALG000000000	51.01	4,861,736	333	29,250
E19	2022-09-02	India: Hyderabad	Environment: water	17.413888°N, 78.473203°E	JAVALF000000000	51.02	5,016,248	293	48,625
E21	2022-09-02	India: Hyderabad	Environment: water	17.414010°N, 78.473498°E	JAVALE000000000	51.1	4,982,495	282	41,246
E22	2022-09-02	India: Hyderabad	Environment: water	17.413863°N, 78.473435°E	JAVALD000000000	51.09	5,003,367	358	32,629
E23	2022-09-02	India: Hyderabad	Environment: water	17.413749°N, 78.473366°E	JAVALC000000000	50.96	4,634,351	260	35,017
E27	2022-09-02	India: Hyderabad	Environment: water	17.4334°N, 78.3869°E	JAVALB000000000	51.05	4,691,292	267	37,034
E29	2022-09-02	India: Hyderabad	Environment: water	17.43283°N, 78.39129°E	JAVALA000000000	51.05	4,875,540	304	37,649
E32	2022-09-02	India: Hyderabad	Environment: water	17.43207°N, 78.390385°E	JAVAKZ000000000	51.2	4,730,639	336	28,338
E38	2022-09-02	India: Hyderabad	Environment: water	17.429899°N, 78.390164°E	JAVAKY000000000	51.15	4,746,047	337	30,343
E39	2022-09-02	India: Hyderabad	Environment: water	17.429576°N, 78.390073°E	JAVAKX000000000	51.08	4,656,254	329	34,307
E45	2022-09-02	India: Hyderabad	Environment: water	17.427748°N, 78.389400°E	JAVAKW000000000	50.85	4,880,668	161	76,280
E48	2022-09-02	India: Hyderabad	Environment: water	17.427768°N, 78.388783°E	JAVAKV000000000	50.93	4,882,237	296	33,426
E51	2022-09-02	India: Hyderabad	Environment: water	17.487778°N, 78.355652°E	JAVAKU000000000	51.06	4,750,050	220	55,655
E52	2022-09-02	India: Hyderabad	Environment: water	17.487409°N, 78.355915°E	JAVAKT000000000	50.91	4,437,703	156	67,233
E53	2022-09-02	India: Hyderabad	Environment: water	17.4888757°N, 78.346739°E	JAVAKS000000000	50.81	4,906,040	116	117,166
E54	2022-09-02	India: Hyderabad	Environment: water	17.483988°N, 78.323579°E	JAVAKR000000000	51.14	4,367,218	251	33,699
E55	2022-09-02	India: Hyderabad	Environment: water	17.483804°N, 78.323011°E	JAVAKQ000000000	51.02	4,682,581	242	40,346
ES03	2022-09-02	India: Hyderabad	Environment: soil	17.437404°N, 78.311776°E	JAVAKP000000000	51.08	4,569,197	261	39,099
ES06	2022-12-18	India: Hyderabad	Environment: soil	17.48169°N, 78.327813°E	JAVAKO000000000	50.94	4,652,033	345	31,813
ES08	2022-12-18	India: Hyderabad	Environment: soil	17.448711°N, 78.309151°E	JAVAKN000000000	50.89	4,873,931	257	38,984
ES11	2022-12-18	India: Hyderabad	Environment: soil	17.432508°N, 78.298304°E	JAVAKM000000000	50.99	4,691,038	223	42,864
ES22	2022-12-18	India: Hyderabad	Environment: soil	17.429877°N, 78.294710°E	JAVAKL000000000	51.12	4,565,870	233	36,527
ES25	2022-12-18	India: Hyderabad	Environment: soil	17.430769°N, 78.300872°E	JAVAKK000000000	50.81	4,787,290	191	57,765
ES26	2022-12-18	India: Hyderabad	Environment: soil	17.430769°N, 78.300872°E	JAVAKJ000000000	51.16	4,561,955	255	37,679
ES29	2022-12-18	India: Hyderabad	Environment: soil	17.430769°N, 78.300872°E	JAVAKI000000000	50.77	4,701,937	163	60,794
ES31	2022-12-18	India: Hyderabad	Environment: soil	17.430769°N, 78.300872°E	JAVAKH000000000	51.09	4,581,022	282	37,545
ES34	2022-12-18	India: Hyderabad	Environment: soil	17.46560°N, 78.3153921°E	JAVAKG000000000	50.73	5,058,848	234	55,349

### DNA fingerprinting

**Fig 2:**
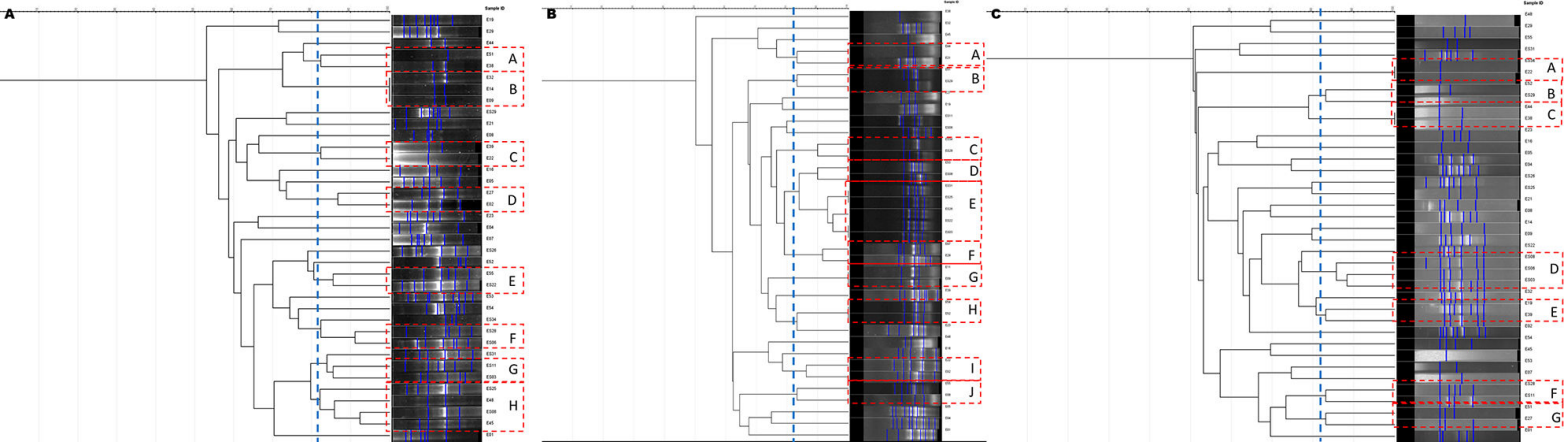
Dendrogram based on phylogenetic analysis of 37 environmental *E. coli* isolates using (A) ERIC-PCR, (**B**) REP-PCR, and (**C**) RAPD-PCR banding analysis by GelJ (29). Dotted boxes represent clusters in which most of them are segregated indicating clonality.

ERIC-PCR of 37 environmental isolates reflected a high degree of clonality within our isolates. At 82% similarity, 19 isolates were grouped into eight small clusters (A–H) according to the band pattern (Fig. 2A). Clustering was observed among isolates of the same phylogroup indicating the genetic similarity among them. Upon REP-PCR, 23 isolates formed 10 small clusters (A–J) (Fig. 2B) and seven small clusters (A–G) were observed based on RAPD-PCR (Fig. 2C) according to the band pattern representing 15 of the 37 isolates. Overall, we observed a high degree of clonality among *E. coli* isolates of phylogroup B1 irrespective of the sample source.

### Biofilm formation ability

**Fig 3:**
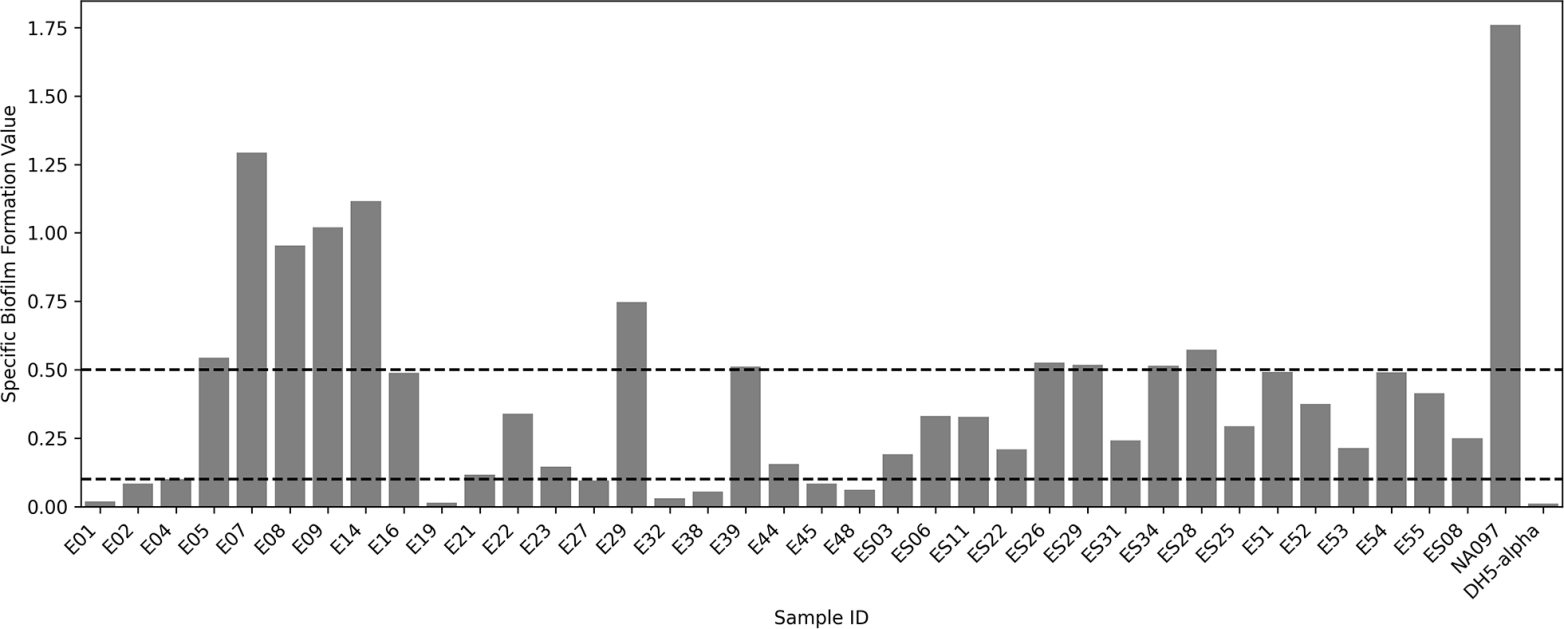
Graphical representation of specific biofilm formation value of each of the isolates. NA097 served as a positive control while DH5α was used as a negative control. While the isolates showing an SBF value of 0.5 or more were considered as strong biofilm formers, those with SBF value less than 0.1 were graded weak biofilm formers; those having SBF value between 0.1 and 0.5 were moderate biofilm formers.

All the isolates displayed biofilm formation capability. Isolates were categorized as strong biofilm formers [specific biofilm formation (SBF) ≥ 0.5], weak biofilm formers (SBF ≤ 0.1), and moderate biofilm formers (SBF ≥ 0.1 to ≤ 0.5). Accordingly, 11 (29.7%) isolates were found to be strong biofilm formers, while 18 (48.6%) were moderate and 8 were weak biofilm formers. Among the MDR isolates, E05 (phylogroup E), E09 (phylogroup B1), and ES26 (phylogroup B1) showed higher SBF values indicating that biofilm formation could possibly contribute to their persistence in the environment (Fig. 3). The potential of biofilm formation may offer an advantage to becoming resistant to multiple antibiotics.

### Comparative genomics

**Fig 4:**
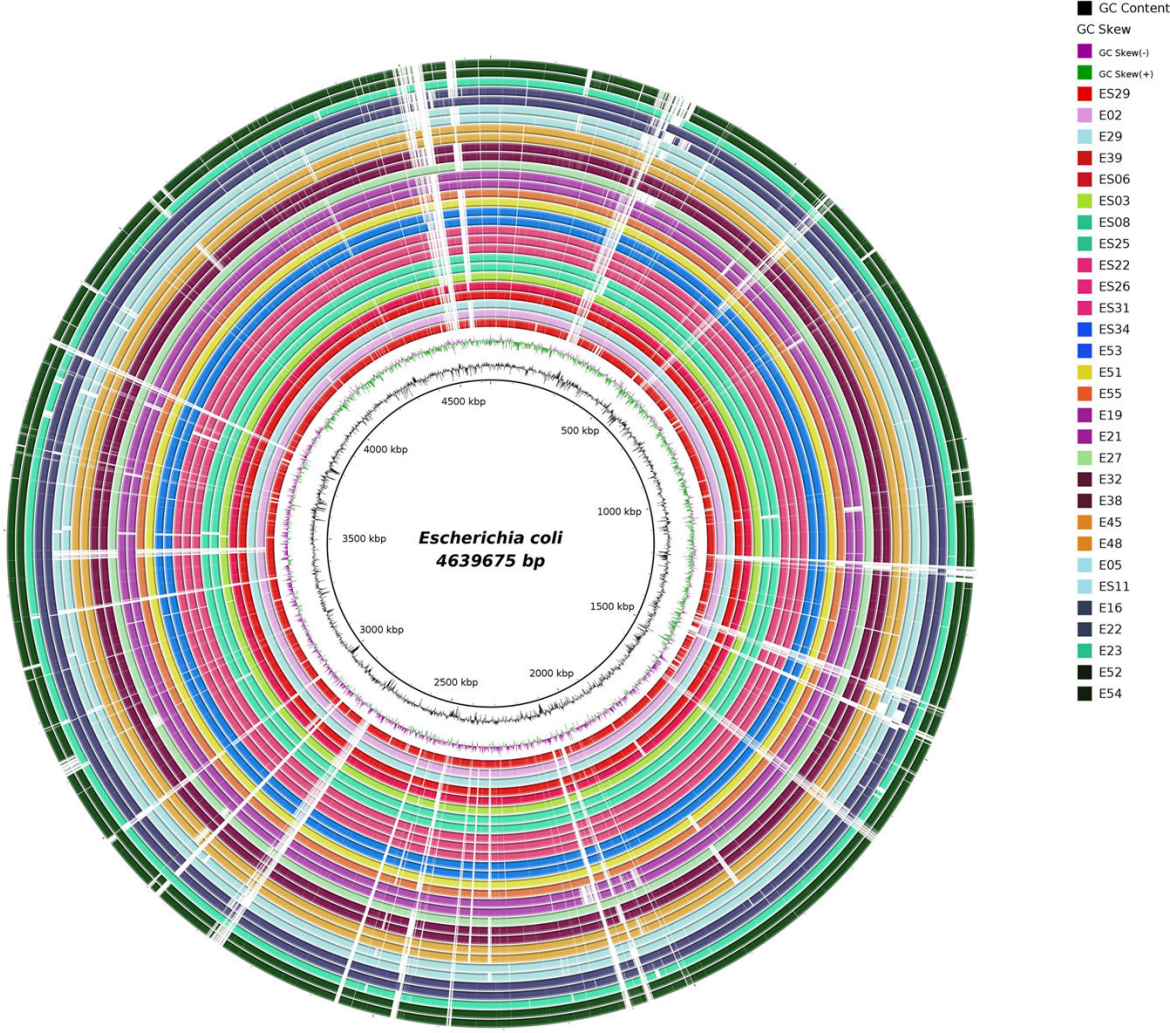
Whole genome comparative analysis of 29 environmental *E. coli* isolates using BRIG, with *E. coli* str. K-12 substr. MG1655 as the reference. Each ring represents a genome, and the rings were color-coded based on their ST, and the genome names were labeled.

**Fig 5:**
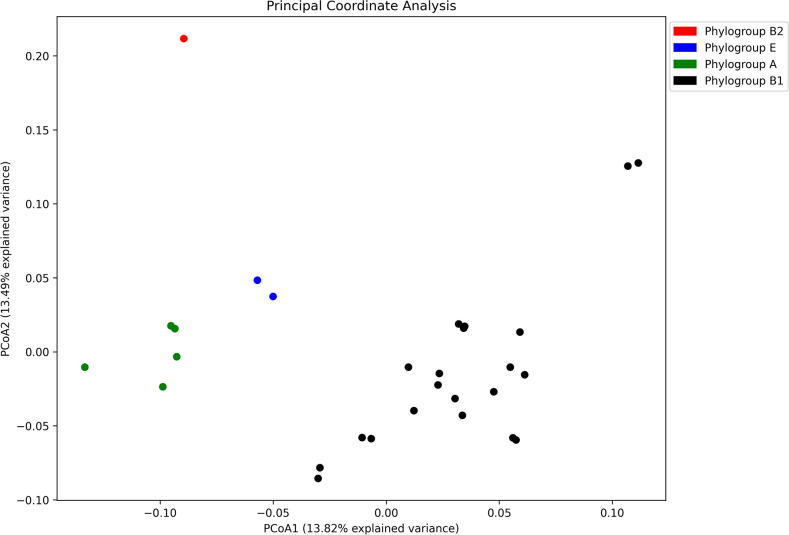
Principal coordinate analysis plot based on the presence/absence binary matrix of the pan genome reflecting the clonality among the isolates belonging to the same phylogroup, irrespective of their source. The isolates have been color-coded according to their respective phylogroups.

Based on quality assessment report obtained from QUAST, 29 of the 37 isolates were considered for further downstream analyses (Table 2). The average genome length of the isolates was observed to be 4.7 Mbp with approximately 51% average GC content. In total, 17 different sequence types (STs) were inferred based on multilocus sequence typing (MLST). Pan genome analysis indicated the presence of 3,064 core genes (99%–100% of isolates), 236 soft core genes (95%–99% of isolates), 1,581 shell genes (15%–95% of isolates), and 6,084 cloud genes (≤15% of the isolates). A total of 6,166 orthogroups and 125,789 genes in orthogroups (99%) were predicted. A total of 23 distinct functional families were established to categorize them. These families encompassed various functions - including cell wall/membrane/envelope biogenesis (*n* = 7,942); translation, ribosomal structure and biogenesis (*n* = 7,572); transcription (*n* = 8,170); replication, recombination, and repair (*n* = 4,631); cell division, cell cycle control, and chromosome partitioning (*n* = 1,419); signal transduction mechanisms (*n* = 4,717); cell motility (*n* = 3,314); extracellular structures (*n* = 364); post-translational modification/protein turnover and chaperones (*n* = 4,468); RNA processing and modification (*n* = 68); mobilome, prophages, transposons (*n* = 1,979); energy production and conversion (*n* = 8,320); carbohydrate transport and metabolism (*n* = 11,286); amino acid transport and metabolism (*n* = 10,127); nucleotide metabolism and transport (*n* = 3,174); intracellular trafficking, secretion, and vesicular transport (*n* = 1,688); coenzyme transport and metabolism (*n* = 5,161); defense mechanisms (*n* = 2,722); lipid transport and metabolism (*n* = 3,738); inorganic ion transport and metabolism (*n* = 5,665); and secondary metabolite biosynthesis, transport, and catabolism (*n* = 1,483) including general functions (*n* = 4,506) with the remaining functional categories classified as unknown. Blast Ring Image Generator (BRIG) analysis indicated that the genomes of these environmental isolates carry similar genomic characteristics while the variable regions were mostly identified as MGEs (Fig. 4). Additionally, principal coordinate analysis (PCoA) showed that isolates belonging to the same phylogroup tend to cluster together irrespective of their source (Fig. 5).

**TABLE 2:** Characterization of isolates according to phylogroup, H type, and ST along with the number of AMR genes, virulence genes, and plasmids

Isolates	Genome accession	Phylogroup	ST	AMR genes	Virulence factors	Plasmids	H type
E02	JAVALI000000000	B1	2165	44	68	1	H7
E05	JAVALH000000000	E	2792	50	79	0	H34
E16	JAVALG000000000	A	218	45	65	1	H30
E22	JAVALD000000000	A	218	43	66	7	H30
E23	JAVALC000000000	A	216	45	49	6	H43
E29	JAVALA000000000	B1	443	47	88	11	H4
E39	JAVAKX000000000	B1	58	51	74	3	H25
ES03	JAVAKP000000000	B1	2005	45	78	0	H19
ES06	JAVAKO000000000	B1	58	48	55	11	H10
ES08	JAVAKN000000000	B1	3863	44	52	7	H10
ES11	JAVAKM000000000	E	–	46	86	0	H25
ES22	JAVAKL000000000	B1	4682	44	78	0	H31
ES25	JAVAKK000000000	B1	3863	45	54	6	H31
ES26	JAVAKJ000000000	B1	4682	44	78	0	H7
ES31	JAVAKH000000000	B1	4682	44	75	0	H5
ES34	JAVAKG000000000	B1	2144	46	77	2	H14
E51	JAVAKU000000000	B1	1727	45	77	4	H5
E52	JAVAKT000000000	A	206	45	46	0	H7
E53	JAVAKS000000000	B1	2144	45	80	7	H7
E54	JAVAKR000000000	A	206	43	43	14	H21
E55	JAVAKQ000000000	B1	196	45	70	14	H10
E19	JAVALF000000000	B1	224	47	90	21	H20
E21	JAVALE000000000	B1	224	47	89	21	H28
E27	JAVALB000000000	B1	12714	52	61	3	H10
E32	JAVAKZ000000000	B1	1258	44	77	10	H28
E38	JAVAKY000000000	B1	1258	45	78	11	–^*a*^
E45	JAVAKW000000000	B1	9960	45	94	4	H5
E48	JAVAKV000000000	B1	9960	45	92	5	H28
ES29	JAVAKI000000000	B2	1236	44	84	4	–

^*a*^ –, denotes indeterminate status.

### *In silico* resistome and virulome profiling

**Fig 6:**
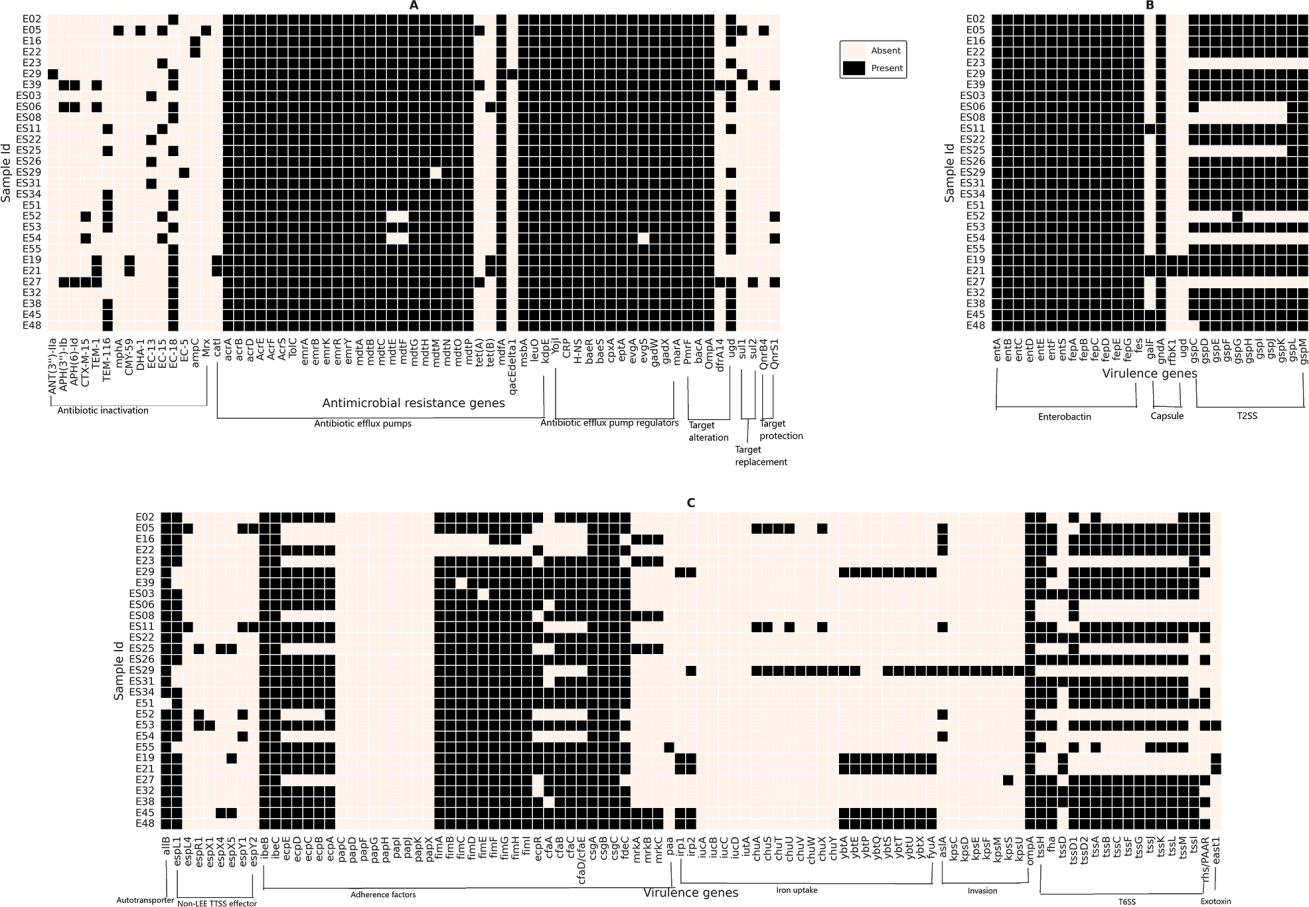
Whole genome-based prediction of (A) antimicrobial resistance and (**B and C**) virulence determinants of 29 environmental isolates using the Comprehensive Antibiotic Resistance Database (32) and Virulence factor database (33), respectively.

WGS-based resistome profiling of the isolates indicated the prevalence of a wide variety of AMR genes, which could be associated with MDR phenotypes among environmental *E. coli*. A combination of resistance genes involved in antibiotic inactivation (*n* = 16), antibiotic efflux pumps (*n* = 31), antibiotic efflux pump regulation (*n* = 11), target alteration (*n* = 5), target protection (*n* = 2), and target replacement (*n* = 2) was predicted in this study as depicted in the heatmap (Fig. 6A). It was observed that genes encoding AcrAB-TolC efflux pump systems, namely, *acrA*, *acrB*, *tolC,* and *acrD,* were conserved across all the isolates. These genes have been previously reported to play an important role in imparting drug resistance, specifically carbapenem resistance (30). Tetracycline resistance encoding genes (*tetA* and *tetB*) were predicted in seven isolates, i.e., *tetA* in E27, E39, E04, and E05 while *tetB* in E19, E21, and ES06 (Fig. 6A), which my be interpreted in line with the results obtained from AST profiling (Fig. 1). All of them, except E05 (phylogroup E), belonged to phylogroup B1. *qnrB4*, a plasmid-associated gene known to impart quinolone resistance (31), was exclusively observed in E05 (phylogroup E).

The *in silico* virulome profile also revealed that environmental isolates are significantly enriched with virulence factors, involved in adherence (*n* = 38), invasion (*n* = 9), auto-transporter activity (*n* = 1), iron uptake (*n* = 24), toxins (*n* = 15), secretion systems [T2SS (*n* = 11), T6SS (*n* = 16), and non-LEE-encoded TTSS (*n* = 8)], and capsule (*n* = 4) (Fig. 6B). The extensive prevalence of the virulence factors may be attributed to the increased virulence capability of the bacteria and ability to surpass host/niche-specific vulnerabilities.

### WGS-based mobile genetic element analysis

**Fig 7:**
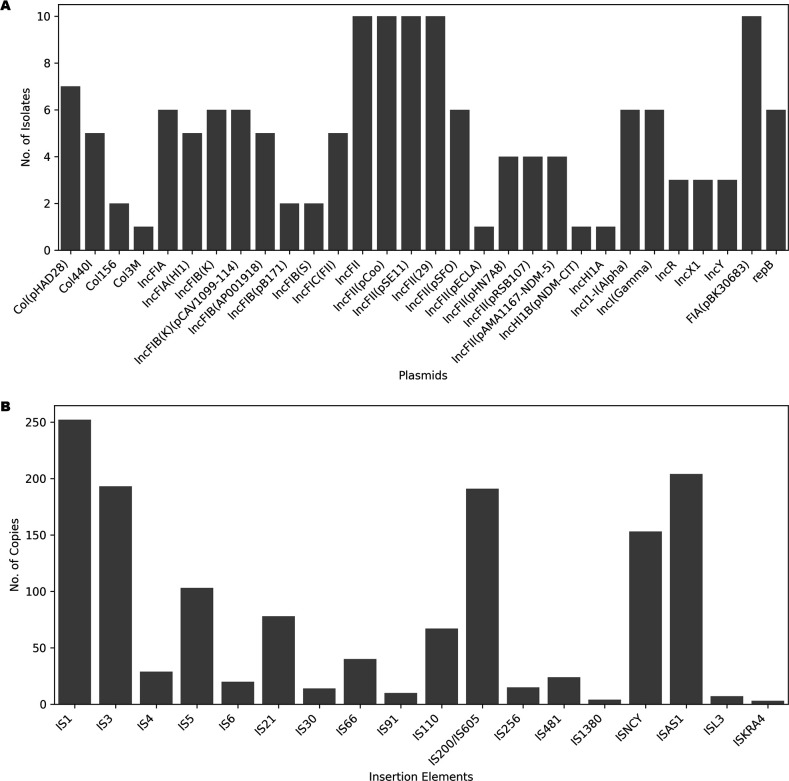
Graphical representation of the occurrence and frequency of mobile genetic elements among the isolates: (A) plasmids obtained using BacAnt (34); (**B**) insertion elements obtained using ISEScan (35). IS1 (252 out of total 1,407 IS copies) was found to be maximally present while ISKRA4 (3 out of 1,407 IS copies) was found to be the least frequent.

A WGS-based prediction of plasmids revealed the predominance of the IncF group, specifically IncFII among isolates under study. Other groups such as IncX1, Col (pHAD28), and Col440I were observed to be less prevalent (Fig. 7A). One of the most important findings of the study was the presence of plasmids harboring carbapenem resistance gene *bla*_NDM-5_. Imipenem resistance inferred from Kirby–Bauer disc diffusion tests also aligned with these genomic findings. Similarly, ES34 was observed to harbor plasmids containing a New Delhi metallo-β-lactamase (NDM) gene named IncHI1B (pNDM-CIT). This finding signifies the potential of HGT to disseminate carbapenem resistance genes among different bacterial isolates. Collectively, these findings emphasize the pivotal role of plasmids, especially those from the IncFII group, in mediating the spread of AMR genes, which has implications for public health in urban settings.

A total of 25 prophage regions commonly found among the members of *Enterobacteriaceae* were predicted based on PHASTER. A prophage region of *Shigella* origin named SfII (accession no. NC_021857) was predominantly present in 9 out of 29 isolates. Similarly, a phage region martha 12B12 (accession no. NC_021070) from *Vibrio* was observed in three isolates (ES34, E51, and E55), and phage phiPSA1 (accession no. NC_024365) of *Pseudomonas* was also observed in E02, E05, E23, E32, and E38 isolates. Additionally, ES06 and E55 were observed to be positive for pipolins (2% of total samples collected). Pipolins are known as MGEs capable of encoding their own B-family DNA polymerase (PolB), which has not been reported in the environmental isolates yet. ISEScan-based screening of the genomes displayed the presence of 18 different kinds of insertion elements [copy numbers of IS1 (18%) were observed to be highest, which generate both types of transposition products–cointegrates and simple insertions, followed by ISAs1 (14.5%) (Fig. 7B)] among our bacterial isolates.

## DISCUSSION

The global emergence of AMR in both humans and animals, along with insufficient public awareness, poses a severe concern within the realms of public health and One Health, particularly in nations with limited economic resources. Consequently, resilient pathogens disseminate throughout the broader environment facilitated by genomic alterations and adaptations, underscoring the necessity of adopting a One Health strategy to curtail the progression and dissemination of AMR. Genome modifications driven by HGT and biotic and abiotic processes may directly or indirectly lead to the transition of a typically benign strain into a pathogenic form (10, 11). A number of studies entailing extensive molecular characterization and high-throughput genomics have significantly advanced our understanding of the various evolutionary pathways followed by *E. coli* and other enteric pathogens (9). The primary aim of this study was to investigate and characterize the environmental isolates of *E. coli* from the urban environment of Hyderabad, Telangana State, India, to decipher the survival and fitness strategies followed by bacteria leading to their persistence and transmission in the environmental conditions. When compared to the isolates from the other phylogroups, PCR-based genotyping (ERIC, REP, and RAPD) of the phylogroup B1 isolates from this study revealed a distinct cluster that represented a majority of the isolates. This appears to be a reasonable case for clonal expansion from a common ancestor. Such a clonal nature poses the risk of possibly triggering locally transmitted outbreaks, while it may simultaneously facilitate the development and implementation of efficient control strategies. PCoA-based clustering also supports the clonal nature of the isolates as depicted by the closed clustering patterns of the isolates belonging to the same phylogroup, irrespective of their origin (Fig. 5).

The present findings regarding the prevalence of isolates belonging to phylogroup B1 in the environmental samples are consistent with other findings (36–38). In addition to phylogroup B1, a reduced prevalence of isolates (E05 and ES11) belonging to the phylogroup E was also observed. These isolates may harbor the characteristics of EHEC, EPEC, EIEC, and ETEC, demanding urgent attention towards tracking their epidemiological source and possible routes of transmission (39). Such a conspecific variation could be attributed to changes in climatic conditions, peculiarities of certain geographic regions, feeding and living practices of the communities in the surroundings, etc. (40). Pathogenic *E. coli* strains that cause extraintestinal infections generally belong to phylogroups B2 and D, whereas commensal strains are identified as members of phylogroup A and B1 (41). Our finding reporting the presence of two isolates (E39 collected from a lake and ES06 collected from slum soil) belonging to ST58 from non-human sources is of particular significance. It has been reported that even though ST58 belongs to commensal phylogroup B1, it shares pathogenic characteristics with other ExPEC members and may be found in poultry, human, or swine (42, 43). As ST58 belongs to the ExPEC group, its presence in the environment may be problematic and highlights the need to adopt a One Health approach. Urinary tract infections, neonatal meningitis, and sepsis are among the extraintestinal diseases that can be caused by ExPEC strains in humans (44–47). In addition to ST58, ST224 was also found in two lake water samples (E19 and E21). This particular ST harbors plasmid-encoded *bla*_CTX-M-15_ gene and is considered as a pandemic- or international high-risk clonal lineage along with ST58 (48). Despite being members of the non-pathogenic commensal phylogroup, the isolates showed resistance to many antimicrobial drugs. The presence of several virulence and antibiotic resistance genes in the isolates may be directly or indirectly attributed to the capability of infecting human/animal hosts. The results of the biofilm formation assay added to our understanding of the development of MDR in these environmental isolates due possibly to their planktonic growth as biofilms (16). A study has revealed that the proteins responsible for the synthesis of type-1 fimbriae, which are encoded by the *fim* gene cluster, are critical for biofilm formation because the mutants of all *fim* genes (except *fimE*, *fimG,* and *fimI*) showed no positive results and displayed significant impairments in forming biofilms (49). In our study, the isolates showed the presence of all the genes of the *fim* gene cluster (*fimA, fimB, fimC, fimD, fimE, fimF, fimG, fimH,* and *fimI*). The moderate biofilm formation ability observed in our isolates could be attributed to the known inhibitory effect of *fimG* product on pilus polymerization (50) and the negative regulatory role of *fimE* in fimbriae synthesis (51) that counteracts with the other genes of the cluster that promotes adherence. Another set of virulence genes, found in our isolates, and involved in adherence was the *csg* gene cluster. The *csgBA* operon supports curli formation in *E. coli* by virtue of two gene products - CsgA, a structural protein, and CsgB, a nucleator protein (52). Deletion mutations of these genes also displayed impaired biofilm formation (49). Hence, the presence of these genes may confer them the ability to form biofilms and ensure persistence and resilience under vulnerable situations.

Even though efflux pumps are part of general physiology, they are well known as an important mechanism of bacteria that may confer AMR (53). The presence of efflux pumps and their regulatory genes in the environmental isolates may influence the accumulation of drugs inside bacterial cells leading to MDR phenotypes. AcrAB-TolC, EmrAB-TolC, and MdtM are the most crucial types of efflux systems for maintaining *E. coli* in the human gut as they can help in expelling bile salts, mammalian steroids, and various antibiotics from the bacterial cells. Although the AcrAB-TolC system is majorly involved in tetracycline resistance and AcrAD-TolC in aminoglycoside resistance, they also facilitate in providing resistance to a wide variety of other drugs and compounds of different physicochemical properties (54–56). A recent study has demonstrated the role of the AcrAB-TolC system in carbapenem resistance (30). The prevalence of imipenem-resistant phenotypes among environmental isolates substantially demonstrates the circulation of carbapenem-resistant isolates in the environment. The prevalence of IncF plasmids found in the environmental isolates in our study may be directly linked to their contribution to the mobilization of AMR traits (57–61). This is in accordance with previous studies wherein *bla*_NDM-5_ was mainly carried by IncF and IncX3 plasmids in *E. coli* (62, 63). Another soil isolate, ES34 with an intermediate imipenem resistance, showed the presence of IncHI1B plasmid encoding carbapenemase gene (pNDM-CIT) that harbors *bla*_NDM-1_. Due to its adverse effects on healthcare and the economy, the emerging NDM, an acquired class B carbapenemase from *Enterobacteriaceae,* has become a significant issue for global public health (64). The *bla*_NDM-5_ gene, which is majorly reported in clinical settings, has also been reported in our study encompassing environmental isolates from lakes. Based on the above findings, we can infer that the other members of the same phylogroup might possess the capability of acquiring similar traits, thereby evolving into more virulent forms. A recently discovered class of integrative MGEs known as pipolins has been found to be present in many different bacterial phyla (65). Primer-independent PolBs (piPolBs) are known to be the distinguishing characteristic of the pipolins. piPolB-encoding elements are replicative family B DNA polymerases (PolB) with an intrinsic capacity for primer synthesis (65) due to which they are classified as self-synthesizing (or self-replicating) MGEs (66). They appear to be helpful in HGT of virulence and resistance-associated genes, possibly taking place in the environmental isolates. The prevalence of insertion sequences (ISs) among bacterial pathogens may help in the transmission of virulence or AMR traits. Their prevalence is seen in the case of a variety of animal pathogens, including *E. coli* (67, 68). Studies have unveiled an intricate interplay between microbes and the factors leading to compensatory adaptations (69). ISs are one of the most prevalent autonomous transposable elements that play a key role in genetic plasticity, adaptability, and evolution of *E. coli* and other prokaryotes (70). A study found that IS elements, specifically those from the families IS1, IS2, IS5, and IS186, are involved in most large-scale bacterial genome rearrangements (71). We also found the presence of IS1 and IS5 in our isolates, which may have helped with genetic recombination to assist survival in the environment.

In summary, a much-needed genomic portrait of the environmental *E. coli* has been deduced. Their genetic relatedness and diversity were dissected with the help of different typing methods. The occurrence and abundance of virulence genes, AMR-encoding genetic features, and an extensive trajectory of MGEs and insertion sequences point to the possibility of *E. coli*’s exquisite survival and evolution in the niches of soil and water while retaining or even augmenting its pathogenic potential. We are hopeful that the genomic data and analyses as presented herein will be useful in understanding the transmission and evolution of this important pathogen in the context of AMR and One Health and devising strategies to counter the global emergence.

## MATERIALS AND METHODS

### Bacterial isolates

A total of 100 environmental samples were examined in this study, including 60 from surface water and 40 from soil. The samples were collected from community service areas (*n* = 12), natural lakes (*n* = 38), sewage water (*n* = 5), and urban slums (*n* = 45). The samples were initially incubated in Luria–Bertani broth for enrichment followed by the selection of Gram-negative bacteria on MacConkey agar plates. Positive colonies were further plated on eosin methylene blue agar for the specific identification of *E. coli* colonies according to colony morphology and appearance on the medium. The positive isolates obtained from this screening were temporarily frozen at −20°C in 25% glycerol until further use. The isolates were also confirmed using the standard IMViC protocols.

### DNA isolation and phylogrouping using multiplex PCR

The genomic DNA was isolated from each isolate using the QIAamp DNA Mini Kit (Qiagen, USA) as per the manufacturer’s protocol. Additionally, RNase A (8 µL, 10 mg/ml) was added to prevent any RNA contamination and incubated at room temperature for another 10 minutes. Multiplex-PCR amplification of the four gene targets, i.e., *arpA*, TspE4.C2 (partial CDS), *chuA*, and *yjaA,* was employed to perform the phylogrouping of the positive environmental isolates using the protocol described earlier (72, 73). The PCR products were then analyzed and visualized using agarose gel electrophoresis with 1.5% agarose gel at 100 V for 2 hours. Based on the presence of genes as visible bands, the isolates were grouped into one of the eight phylogroups named as A, B1, B2, C, D, E, and F and *Escherichia* cryptic clade I according to the scheme described previously (72).

### Molecular typing using different DNA fingerprinting techniques

The fingerprint analysis using ERIC-PCR was carried out, as previously mentioned (74). Similarly, REP-PCR and RAPD-PCR were performed according to the established protocols (75). PCR amplifications were carried out at specified reaction conditions for 30 cycles each, as described earlier (44, 73). GelJ software (29) was used to compare isolate-specific DNA banding profiles obtained on a 1.5% agarose gel for each method. Dendrograms were obtained by the dice similarity index based on the unweighted pair group method with arithmetic mean algorithm to examine similarity and diversity within these environmental isolates.

### Antimicrobial susceptibility testing

Isolates were subjected to AST using the Kirby–Bauer disc diffusion method on bacterial lawns cultured on Mueller–Hinton agar plates. Antibiotic discs (HiMedia, India) for 16 different antibiotics, i.e., clarithromycin (15 µg), chloramphenicol (30 µg), fosfomycin (200 µg), tetracycline (30 µg), co-trimoxazole (25 µg), gentamicin (10 µg), doxycycline (30 µg), nalidixic acid (30 µg), nitrofurantoin (30 µg), cefotaxime (30 µg), cefazolin (30 µg), aztreonam (30 µg), ampicillin (10 µg), imipenem (10 µg), ampicillin/sulbactam (10/10 µg), and ciprofloxacin (5 µg) belonging to eight different classes, were used for AST of the isolates. After incubation, the zone of inhibition around each disc was measured using Zone Scales (HiMedia), and the status was assigned according to the guidelines laid by the CLSI (2021). Isolates that demonstrated resistance against three or more classes of antimicrobial drugs were categorized to be MDR.

### Biofilm formation assay

Using a previously described method (76), the biofilm formation capacity of the isolates was examined by performing the biofilm formation assay twice in technical triplicates. Briefly, the overnight grown bacterial cultures were diluted to obtain an OD of 0.05 in a fresh minimal medium (M63), 200 µL of which was pipetted in triplicates into the sterile 96-well microtiter plates. The initial OD at 600 nm (OD_600 (0 h)_) was obtained, and plates were incubated at a stationary condition for 48 hours at 28°C. Afterward, OD was measured at 600 nm (OD_600 (48 h)_) followed by washing and fixing of the cells. Further, the cells were stained using 0.1% crystal violet solution for 30 minutes followed by solubilization of attached cells with 300 µL of a solution containing ethanol:acetone (80:20). The OD of solubilized cells was measured at 570 nm. Further, the SBF value was calculated using the formula as used/described earlier (76).

### WGS, assembly, and annotation

WGS of the positive isolates was carried out on an Illumina MiSeq system. The paired-end sequence reads were filtered and trimmed using the FastQC version 0.12.0 (77) as per the Phred score cut-off value of 33. The high-quality reads were subjected to *de novo* assembly using SPAdes Genome Assembler (v3.15.4) (78) followed by quality assessment using QUAST (79) and CheckM (80) and annotation using PROKKA (81). Further, pan genome analysis was done using Roary (82). Principal coordinate analysis was performed using in-house Python scripts with the gene presence/absence data obtained from pan genome analysis. This was followed by the identification of sequence types using the *in silico* MLST pipeline. Since *E. coli* is distributed among eight different phylogroups, ClermonTyping was used for phylotyping of the isolates (83). Further, OrthoFinder (84) was used to predict the orthologs present in the isolates, and the orthogroups were classified according to their function using COGclassifier (v1.0.5) (85). *E. coli* K-12 substr. MG1655 complete genome was used as the reference genome for the analysis using Blast Ring Image Generator (BRIG-0.95-dist) wherein the genomes were compared to the reference to determine their genetic relatedness (86).

### Whole genome-based analysis of resistome, virulome, and mobilome

Resistome and virulome profiling of the genomes under study was done using ABRicate (v1.0.1) that performs BLAST (v2.13.0) against the Comprehensive Antibiotic Resistance Database (32) and the Virulence factor database (33), respectively. The threshold parameters for both were percentage identity >80% and percentage coverage >95%. Heatmaps depicting the presence–absence status of the virulence and antimicrobial resistance genes were plotted using matplotlib and seaborn libraries of Python (32, 33, 87).

In our study, the prediction of plasmids, prophage regions, and insertion elements was done using bioinformatics tools. Plasmid sequences were identified using the PlasmidFinder (88) database with BacAnt (34). Further, the genomes were also screened for the presence of newly discovered self-synthesizing transposons known as pipolins using the method described previously (89), followed by prophage element prediction using PHASTER (PHAge Search Tool Enhanced Release) (90). ISEScan (35), a tool for the identification of insertion sequences, was used to predict the presence of insertion sequences in the genomes, which may highlight the adaptability of the bacteria to certain conditions and acquisition of complementing traits. Results were analyzed using in-house written Python scripts tailored to handle the genomes of bacterial isolates.
